# Assessing sirtuin expression in endometrial carcinoma and non-neoplastic endometrium

**DOI:** 10.18632/oncotarget.6691

**Published:** 2015-12-19

**Authors:** Carla Bartosch, Sara Monteiro-Reis, Diogo Almeida-Rios, Renata Vieira, Armando Castro, Manuel Moutinho, Marta Rodrigues, Inês Graça, José Manuel Lopes, Carmen Jerónimo

**Affiliations:** ^1^ Department of Pathology, Portuguese Oncology Institute-Porto (IPO-Porto), Porto, Portugal; ^2^ Cancer Epigenetics & Biology Group, Research Center, Portuguese Oncology Institute-Porto, Porto, Portugal; ^3^ Department of Pathology and Oncology, Medical Faculty, University of Porto, Porto, Portugal; ^4^ Department of Pathology, Centro Hospitalar São João (CHSJ), Porto, Portugal; ^5^ IPATIMUP (Institute of Molecular Pathology and Immunology, University of Porto), Porto, Portugal; ^6^ Department of Pathology and Molecular Immunology, Institute of Biomedical Sciences Abel Salazar (ICBAS), University of Porto, Porto, Portugal

**Keywords:** endometrial carcinoma, endometrium, sirtuin, SIRT1, SIRT7, Pathology Section

## Abstract

Sirtuins participate in hormone imbalance, metabolism and aging, which are important processes for endometrial cancer (EC) development. Sirtuins mRNA expression (SIRT1 to 7) was determined in 76 ECs (63 Type I, 12 Type II and one mixed EC), and 30 non-neoplastic endometria (NNE) by quantitative real-time PCR. SIRT1 and SIRT7 protein expression was evaluated by immunohistochemistry using Allred score. Compared to NNE, ECs showed SIRT7 (*p* < 0.001) mRNA overexpression, whereas SIRT1 (*p* < 0.001), SIRT2 (*p* < 0.001), SIRT4 (*p* < 0.001) and SIRT5 (*p* < 0.001) were underexpressed. No significant differences were observed for SIRT3 and SIRT6. Type II ECs displayed lower SIRT1 (*p* = 0.032) and SIRT3 (*p* = 0.016) transcript levels than Type I ECs. Concerning protein expression, SIRT1 immunostaining median score was higher in ECs compared to NNE epithelium (EC = 5 *vs*. NNE = 2, *p* < 0.001), while SIRT7 was lower in ECs (EC = 6 *vs*. NNE = 7, *p* < 0.001). No significant associations were found between SIRT1/7 immunoexpression and histological subtype, grade, lymphovascular invasion or stage. Our data shows that sirtuins are deregulated in EC. The diversity of expression patterns observed suggests that sirtuins may have distinctive roles in endometrial cancer similarly to what has been described in other cancer models.

## INTRODUCTION

The majority of endometrial carcinomas (ECs) are endometrioid, that is, Type I and result from estrogenic stimulation, being associated with risk factors like anovulation, nulliparity, unopposed estrogen therapy and tamoxifen [1]. Furthermore, Type I ECs are also associated with risk factors, such as obesity, diabetes and hypertension that are part of a clinical condition known as metabolic syndrome [2]. On the other hand, Type II carcinomas are not related to estrogen excess and usually occur at an older age. Approximately 10% of ECs are Type II carcinomas, including serous and clear cell carcinomas.

Sirtuins are a family of NAD(+)-dependent deacetylases that participate in the regulation of metabolism, cell division and aging [3]. Their role in cancer is largely unknown, but seems to be complex, as sirtuins apparently may work both as oncogenes and tumor suppressors [4-7]. Indeed, sirtuins participate in DNA repair, genomic stability maintenance and replicative life span control, thus their functional loss may promote tumorigenesis. Conversely, their presence is essential for cancer metabolic reprogramming, allowing tumor growth and survival under stress conditions.

Currently, limited data has been published regarding sirtuins role in endometrium pathology. Two studies suggested that SIRT1 may promote endometrial tumor growth [8, 9], while others described its potential role in endometriosis [10] and embryo endometrial receptivity [11].

Given the link between metabolism, aging and tumorigenesis in EC, sirtuins pose as excellent candidates as participants in EC development. Thus, in this study we aimed to characterize the expression of sirtuins in EC comparing with non-neoplastic endometrium (NNE).

## RESULTS

The clinico-pathological features of our series of EC patients are described in Table 1. The mean age at diagnosis was 67 years, ranging from 39 to 88 years. All patients underwent total hysterectomy, 29 (38.2%) with lymphadenectomy. Frozen section was performed in 42 (55.3%).

**Table 1:** Clinicopathological features of endometrial carcinoma patients included in the study

**Age (years); mean ± SD**	67.0±11.7
**Menopausal status; n (%)**	
Pre-menopause	7 (9.2)
Post-menopause	69 (90.8)
**Surgical procedure; n (%)**	
TH	47 (61.8)
TH+LN	29 (38.2)
**Tumor size (cm); mean ± SD**	4.4±2.2
**Histological subtype; n (%)**	
**Type I**	
- Endometrioid, no specific type	41 (53.9)
- Endometrioid with squamous differentiation	16 (21.1)
- Endometrioid corded and hyalinized	1 (1.3)
- Undifferentiated	1 (1.3
- Dedifferentiated	2 (2.6)
**Type II**	
- Serous	10 (13.2)
- Clear cell	3 (3.9)
Mixed endometrioid and serous	1 (1.3)
Ambiguous	1 (1.3)
**Histological FIGO grade; n (%)**	
Grade 1	24 (41.4)
Grade 2	21 (36.2)
Grade 3	13 (22.4)
**Myometrial invasion; n (%)**	
None or <50%	50 (65.8)
≥ 50%	26 (34.2)
**Cervical invasion; n (%)**	
None or epithelial	60 (78.9)
Stromal	16 (21.1)
**Lymphovascular invasion; n (%)**	
Present	26 (34.2)
Absent	50 (65.8)
**FIGO stage; n (%)**	
I	51 (67.1)
II	9 (11.8)
III	11 (14.5)
IV	5 (6.6)

FIGO - International Federation of Gynecology and Obstetrics;

LN – Lymphadenectomy; SD – Standard deviation; TH – Total hysterectomy.

Most were Type I EC, including endometrioid carcinomas (*n* = 58, 76.3%), undifferentiated (*n* = 1, 1.3%) and dedifferentiated carcinomas (*n* = 2, 2.6%). Type II carcinomas included serous (*n* = 10, 13.2%) and clear cell carcinomas (*n* = 3, 3.9%). There was one carcinoma with ambiguous features and one mixed endometrioid and serous carcinoma. Endometrioid carcinomas were classified as well (*n* = 24, 41.4%), moderately (*n* = 21, 36.2%) or poorly (*n* = 13, 22.4%) differentiated. The majority did not invade more than half of the myometrium (*n* = 50, 65.8%) nor involved the endocervical stroma (*n* = 60, 78.9%). Lymphovascular invasion was identified in 26 (34.2%).

The majority of patients had disease limited to the uterus at diagnosis (*n* = 60, 78.9%). Sixteen (21.1%) presented with extra-uterine disease, including lymph node metastases (*n* = 7, 9.2%) and peritoneal or distant metastases (*n* = 5, 6.5%). Six patients developed recurrences. Six patients died of EC and six died of other causes. The median follow-up time for survivors was 18.3 months (range: 4.8 to 70.3 months), with 10 (15.4%) patients followed for at least 5 or more years after primary diagnosis.

The NNE samples were collected from hysterectomy specimens of patients diagnosed with leiomyoma (*n* = 12, 40.0%), endometrial polyp (*n* = 3, 10%), uterine prolapse (*n* = 2, 6.7%) and benign ovarian lesions (*n* = 13, 43.3%). The mean age of these patients was 63.1 years (SD: ± 9.4). The majority consisted of atrophic or inactive endometrium (*n* = 27, 86.7%), and four (13.3%) were classified as proliferative.

**Figure 1:**
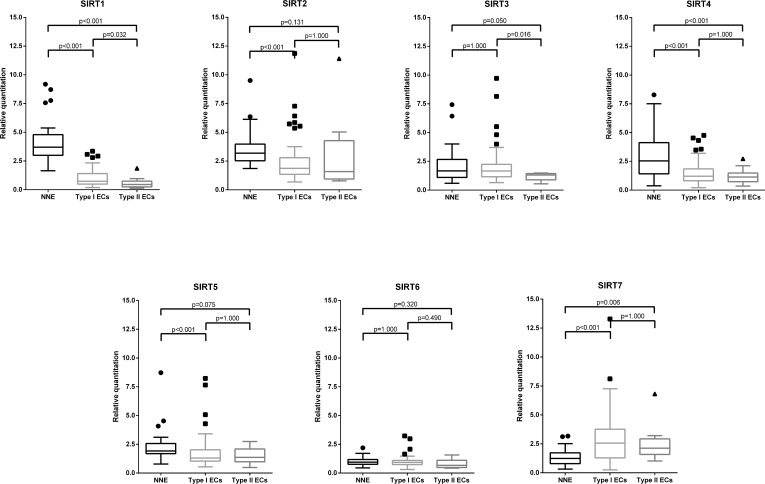
Sirtuin mRNA expression: Box-plots comparing expression levels between endometrial carcinomas (EC) histological types and non-neoplastic endometria (NNE)

Expression levels of sirtuin mRNA, evaluated by qRT-PCR, varied among ECs and NNE. SIRT1, SIRT2, SIRT4 and SIRT5 were significantly underexpressed (all *p* < 0.001) in ECs compared to NNE samples, whereas SIRT7 was overexpressed (*p* < 0.001). No significant differences were observed between the two groups for SIRT3 (*p* = 0.466) and SIRT6 (*p* = 0.447) expression levels. Regarding, EC types, SIRT1 and SIRT3 were significantly overexpressed in Type I compared to Type II EC (Figure 1). No significant differences were found within EC types for the other sirtuins.

**Figure 2:**
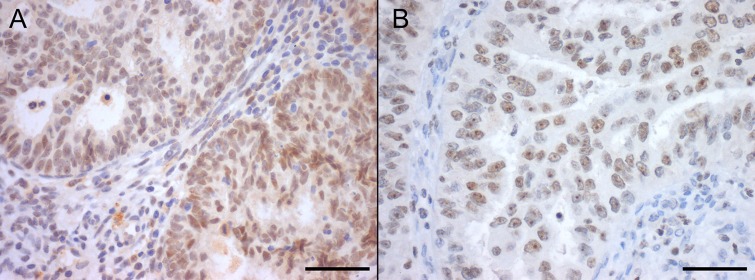
Nuclear SIRT1 (A) and SIRT7 (B) protein immunoexpression in endometrial carcinomas, with marked nucleolar staining in SIRT7 (Bar = 50 μm)

**Figure 3:**
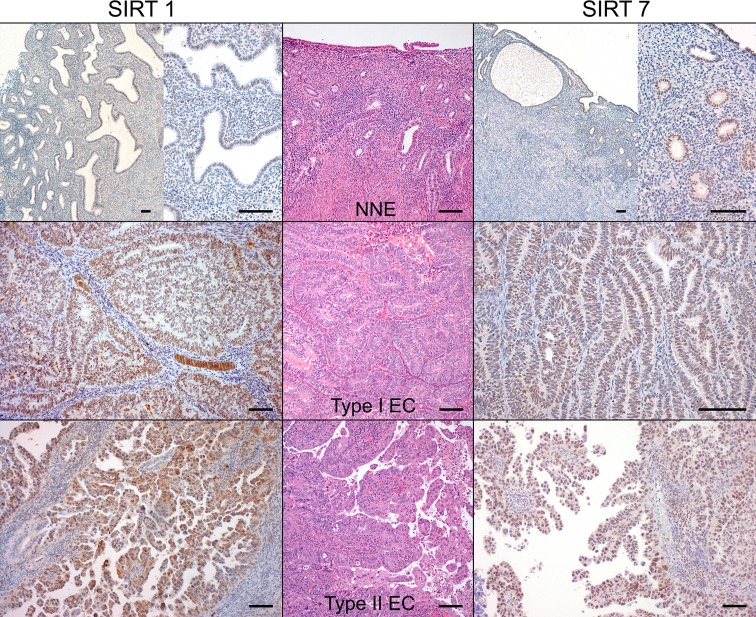
SIRT1 and SIRT7 protein immunoexpression in endometrial carcinomas and non-neoplastic endometria (Bar = 100 μm)

Since striking differences were observed for SIRT1 and SIRT7 transcript levels, we further analyzed their protein expression by immunohistochemistry (IHC) and Western-blot. SIRT1 IHC expression, when present, was predominantly nuclear and homogeneous both in ECs and NNE epithelium (Figure 2A). There was a significant higher proportion of SIRT1 positive cases of ECs (*n* = 70, 92.1%) compared to NNE (*n* = 13, 43.3%, *p* < 0.001). Indeed, a significantly higher nuclear staining score was found in ECs compared to NNE epithelium (median score: EC = 5 *vs*. NNE = 2, *p* < 0.001) (Figure 3). Weak cytoplasmic staining was also observed in NNE epithelium, while in ECs focal cytoplasmic staining was rarely observed. Endometrial stroma and myometrium showed weak, patchy nuclear and cytoplasmic staining.

SIRT7 IHC expression was localized to the nucleus and presented either as homogeneous or nucleolar as shown in Figure 2B. The proportion of SIRT7 positive cases in ECs (*n* = 73, 96.1%) was slightly smaller than in NNE (*n* = 30, 100%, *p* = 0.557). However, a significant lower staining score was observed in ECs compared to NNE epithelium (median score: EC = 6 *vs*. NNE = 7, *p* < 0.001) (Figure 3). Of notice, SIRT7 expression in ECs was highly variable ranging from score 2 to 8, while in NNE epithelium the expression was quite consistent, ranging from score 6 to 8. Weak cytoplasmic staining was observed in ECs and NNE epithelium. Lymphocytes showed strong homogenous nuclear SIRT7 staining and were used as internal positive control. Endometrial stroma and myometrium were negative.

Moreover, no significant differences were found between tumor types for both SIRT1 and SIRT7 protein immunoexpression. Similarly, no association was established with any of the clinico-pathological variables, including grade, lymphovascular invasion and FIGO stage (Table 2).

**Table 2:** Association of SIRT1 and SIRT7 protein expression and clinicopathological features

*SIRT1*	*Allred Score (n)*								*p*
	0	2	3	4	5	6	7	8	
Endometrial carcinomas*	**0**	**5**	**9**	**15**	**18**	**18**	**8**	**2**	
Histological subtype**									***0.913***
*Type I*	0	4	7	14	14	16	6	1	
*Type II*	0	1	3	1	4	2	1	1	
***Histological FIGO grade***									***0.572***
*Grade 1*	0	1	2	6	4	7	3	0	
*Grade 2*	0	2	3	4	7	4	0	1	
*Grade 3*	0	1	1	4	2	3	2	0	
***Myometrial invasion***									***0.670***
*None or <50%*	0	3	6	11	13	8	7	1	
*≥ 50%*	0	2	3	4	5	10	1	1	
***Cervical invasion***									***0.460***
*None or epithelial*	0	4	7	14	13	13	7	1	
*Stromal*	0	1	2	1	5	5	1	1	
***Lymphovascular invasion***									***0.964***
*Present*	0	3	5	11	13	10	6	1	
*Absent*	0	2	4	4	5	8	2	1	
***FIGO stage***									***0.846***
*I*	0	3	6	11	11	11	7	1	
*II/III/IV*	0	2	3	4	7	7	1	1	
***Non-neoplastic endometrium***	**4**	**13**	**4**	**3**	**4**	**1**	**1**	**0**	

*SIRT7*	*Allred Score (n)*								*P*
	0	2	3	4	5	6	7	8	
***Endometrial carcinomas***	**0**	**3**	**1**	**6**	**14**	**20**	**19**	**13**	
Histological subtype**									***0.973***
*Type I*	0	2	1	4	12	17	18	9	
*Type II*	0	1	0	2	2	3	2	4	
***Histological FIGO grade***									***0.476***
*Grade 1*	0	0	0	2	5	8	3	6	
*Grade 2*	0	0	0	2	4	4	10	1	
*Grade 3*	0	2	1	0	2	4	3	1	
***Myometrial invasion***									***0.199***
*None or <50%*	0	2	1	3	9	11	13	11	
*≥ 50%*	0	1	0	3	5	9	6	2	
***Cervical invasion***									***0.667***
*None or epithelial*	0	2	1	4	11	17	14	11	
*Stromal*	0	1	0	2	3	3	5	2	
***Lymphovascular invasion***									***0.583***
*Present*	0	3	0	5	10	11	12	9	
*Absent*	0	0	1	1	4	9	7	4	
***FIGO stage***									***0.359***
*I*	0	2	1	4	8	13	12	11	
*II/III/IV*	0	1	0	2	6	7	7	2	
***Non-neoplastic endometrium***	**0**	**0**	**0**	**0**	**0**	**8**	**10**	**12**	

* SIRT1 data was not available in one case.

** For the purpose of this analysis the two components of the mixed endometrioid and serous carcinoma were evaluated separately, and the ambiguous carcinoma was included in the Type I group.

FIGO - International Federation of Gynecology and Obstetrics.

**Figure 4:**
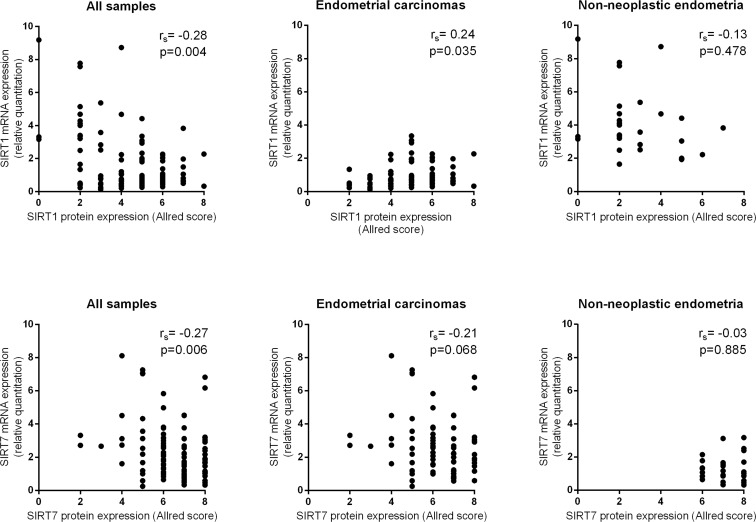
Scatter plots showing SIRT 1 and SIRT7 protein and mRNA expression correlation

Globally, both SIRT1 (r_s_ = −0.28, *p* = 0.004) and SIRT7 (r_s_ = −0.27, *p* = 0.006) protein IHC expression were inversely correlated with mRNA expression (Figure 4). When analyzing the results separately for ECs and NNE, a positive correlation for SIRT1 mRNA and protein IHC expression was found in ECs (r_s_ = 0.24, *p* = 0.035), whereas no significant correlation was found in NNE (r_s_ = −0.13, *p* = 0.478). Conversely, no significant correlation was found for SIRT7 mRNA and protein IHC expression in ECs (r_s_ = −0.21, *p* = 0.068) or NNE (r_s_ = −0.03, *p* = 0.885).

**Figure 5:**
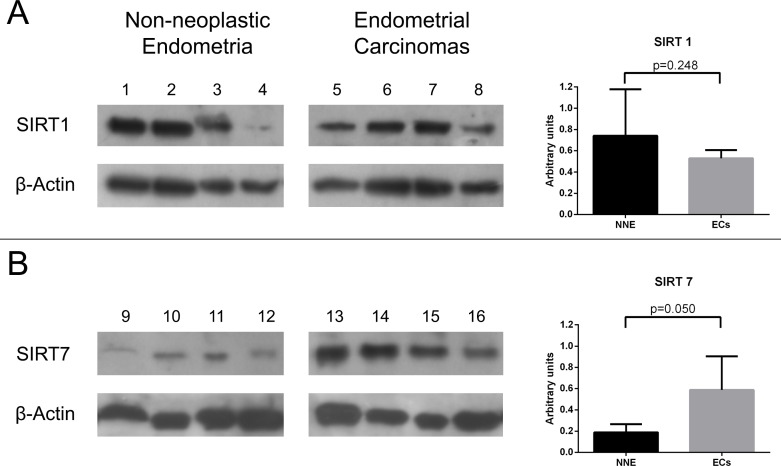
Western-blot analysis for SIRT 1 (A) and SIRT 7 (B) proteins using non-neoplastic endometria (NNE) and endometrial carcinoma (EC) samples The bar graphs on the right represent mean ± standard deviation of relative protein optical density values after normalization for β-Actin loading as described in methods.

For Western-blot analysis we chose cases that showed the most discrepant results between protein IHC expression and mRNA qRT-PCR levels. That is, for SIRT1 western-blot analysis, we included cases of ECs with low mRNA expression but high IHC protein expression, as well as NNE with high mRNA expression but low IHC protein expression. The SIRT1 protein was detected as a band located at ≈ 110 kDa. Both ECs and NNE samples showed similar SIRT1 protein content in densitometry analysis (Figure 5A). For SIRT7 we selected ECs that showed high mRNA expression but low IHC protein expression, and NNE that showed low mRNA but high protein IHC expression. The SIRT7 protein was detected as a single band located at ≈ 45 kDa. EC samples showed higher SIRT7 protein content than NNE samples (Figure 5B).

## DISCUSSION

In this study we performed a general survey on sirtuins (SIRT1- SIRT7) mRNA expression in ECs *vs.* NNE and found significant differences in SIRT1, SIRT2, SIRT4, SIRT5 and SIRT7 mRNA levels. The most striking and interesting differences were observed in SIRT1 and SIRT7, which were, respectively, underexpressed and overexpressed in ECs. Thus, we further assessed SIRT1 and SIRT7 IHC protein expression, and significant differences in Allred score between ECs and NNE were depicted. Interestingly, IHC results were opposite to those of mRNA expression, both for SIRT1 and SIRT7, i.e., higher and lower Allred scores for ECs, respectively.

The recent research on sirtuins in cancer, points toward variability of function depending on tumor type, stage, microenvironment and signaling pathway affected [4-7]. Sirtuins have different enzymatic activities, acting in different directions with cross-talk and feedback regulation between them. Additionally, sirtuins are present in different subcellular locations, including the nucleus, cytoplasm and mitochondria, consistent with their functional diversity.

SIRT1 is the most well studied member of the sirtuin family. It acts primarily as lysine deacetylase and has been reported to have nuclear and cytosolic activities. It is responsible for modifications in histone tails, including H4K16 hypoacetylation, which is associated with tumorigenesis, but also of several other subtracts, including ER, Beta-catenin and p53 which are known key players in EC development [6, 12-16]. A dual function in tumor promoting and suppression has been described for SIRT1, being upregulated in some cancer types and downregulated in others [17, 18].

SIRT7 is a lysine deacetylase that is usually located in the nucleus, predominantly in the nucleolus [19, 20]. SIRT7 has been shown to function as an oncogene, being upregulated in many cancer types [19]. Its enhanced activity seems important to maintain oncogenic properties through H3K18 deacetylation, and also assure rDNA transcription to meet the increased demand of ribosome synthesis in proliferating cancer cells [19]. Additionally it also interacts with p53 and assists SIRT1 in promoting cell migration and invasiveness [21, 22].

Only few studies have described the expression of SIRT1 in ECs, most with limited data. Lin et al. observed that SIRT1 mRNA and protein were overexpressed in EC compared to adjacent “normal” endometrium, and hypothesized that SIRT1 promotes tumor proliferation and invasion by targeting SREBP1 and lipogenesis in EC. Both Guo et al. [23] and Asaka et al. [9] presented data showing SIRT1 protein overexpression in EC compared to “normal” endometrium, while the opposite was observed by Marc et al. (dissertation) [24]. In our study we also observed SIRT1 IHC protein overexpression in EC, while SIRT1 mRNA was underexpressed. Interestingly, the SIRT1 antibody used by Asaka et al. [9] shows a predominantly cytoplasmatic staining, while ours clearly has a predominant nuclear staining, most likely due to different epitope targeting. Importantly, we believe that the evaluation of nuclear expression is more adequate to study the role of sirtuins as histone-modifying enzymes. The conflicting results among studies might also be due to differences in the selection and evaluation of the endometrium samples used as controls. Indeed, in our study most NNE samples were classified as atrophic or inactive; in the study by Marc et al. [24] secretory endometrium was used; in Lin et al. [8] the controls were sampled from tumor-adjacent endometrium; Asaka et al.[9] used proliferative, secretory and atrophic endometrium; and in Guo et al. [23]study the controls were reported as “normal endometrium”, not otherwise specified. The endometrium displays striking differences in terms of proliferation and differentiation during the menstrual cycle and in menopause. No study has fully characterized the expression of sirtuins in different types of endometrium. Asaka et al. suggested a higher expression of SIRT1 in secretory endometrium. Yet, there are studies reporting that other histone modifying enzymes, including some histone deacetylases, are differently expressed throughout the menstrual cycle [25, 26]. Additionally, the potential field effect of alterations must be taken into account when using endometrium adjacent to tumor as a control [27].

Regarding differences between sirtuin mRNA and IHC protein expression found in our study, there are several possible explanatory mechanisms including those responsible for RNA turnover and post-translational control of protein turnover and abundance [28, 29]. However, it is important to note that, in the present study, glandular and stromal endometrial cellular components of NNE displayed different expression. We are aware that, while in qRT-PCR and Western-blot all cells present in the tissue sample were analyzed, in IHC only the epithelial component was scored for comparison with EC, thus potentially explaining the observed differences. This is supported by the results of correlation analysis between mRNA and IHC protein expression in EC and NNE separately. Noteworthy, many studies of endometrial lesions that compare the epigenetic state of lesions with “normal” endometrium give little regard to endometrial cycle stage or cell components, thus potentially biasing their results. Altmäe et al. discussed guidelines for endometrium “omics”, stressing that the intrinsic variability of the endometrium, comprising the different cell types and the dynamic nature of the tissue response to the cyclic hormonal milieu, needs to be considered for adequate design and analysis of endometrial studies [30].

In our study no association was observed between SIRT1/7 protein expression and tumor type, grade, lymphovascular invasion or stage. Marc et al. [24] reports similar results, but Asaka et al. [9] found significantly higher SIRT1 expression in grade 3 tumors and in ECs with lymphovascular invasion. Again, results must be interpreted carefully due to differences in antibodies, IHC scoring method and statistics used.

To the best of our knowledge no study has previously assessed the expression of SIRT7 in endometrial lesions. SIRT7 mRNA is ubiquitously expressed in different human tissues and the level of expression appears to be associated with proliferative activity [20]. This is in accordance with higher SIRT7 mRNA expression observed in ECs compared to NNE, the latter consisting mostly of inactive or atrophic endometrium, thus, with low proliferation activity. Even though SIRT7 IHC evaluation showed a significant lower score for ECs, the difference in median scores is very small and mainly due to a wider range of scores in ECs.

The different sirtuin expression levels between ECs and NNE observed in our study, as well as the divergent results for SIRT1 and SIRT7 expression, despite the limitations discussed, suggest that these enzymes might indeed participate in EC tumorigenesis with putative oncogenic and tumor-suppressive functions as described in other models. Because sirtuin inhibitors are emerging as a promising anti-cancer strategy, it is important to clarify the function of sirtuins in ECs in further studies [31].

## MATERIALS AND METHODS

### Patients and samples

Tissue samples of 76 ECs (63 Type I, 12 Type II and one mixed EC), and 30 non-neoplastic endometria (NNE) were previously freshly collected from hysterectomy specimens and snap-frozen in liquid nitrogen, as part of Centro Hospitalar S. João (CHSJ) and Portuguese Oncology Institute - Porto (IPO-Porto) tumor tissue banks. The two components of the mixed EC were sampled and analyzed separately. Five-micron thick sections were cut in a cryostat and stained to allow the identification of target areas. Subsequently, an average of 15, 10μm thick, sections from each specimen were cut and trimmed to maximize the yield of target cells. At the end, an additional section was also stained to confirm that the representativeness of the tissue was maintained. For each case formalin-fixed and paraffin-embedded (FFPE) samples had also been collected, including twin parallel fragments of the fresh-frozen specimens and adequate samples for routine histopathological examination.

All pathological material was reviewed by a pathologist with experience in gynecopathology (CB) and relevant clinical data was collected from the patient's files. This study was approved by the institutional review boards of both institutions [CHSJ (CES44/2010)/IPOP (CES494-010)].

### Quantitative real-time polymerase chain reaction

Total RNA was extracted from the fresh-frozen samples using Trizol reagent (Invitrogen, Carlsbad, USA). From each sample, 1 μg of total RNA was transcribed into cDNA by reverse transcription using the High Capacity cDNA Reverse Transcription Kit (Applied Biosystems, Foster City, USA), according to the manufacturer's instructions.

Expression levels of SIRT1 to 7 mRNA were determined by quantitative real-time polymerase chain reaction (qRT-PCR) using previously synthesized sample cDNA as template. The following gene expression assays from Applied Biosystems were used: SIRT1 (Hs01009005_m1), SIRT2 (Hs00247263_m1), SIRT3 (Hs00202030_m1), SIRT4 (Hs00202033_m1), SIRT5 (Hs80978535_m1), SIRT6 (Hs00213036_m1) and SIRT7 (Hs01034735_m1). The qRT-PCR was performed in a 10μl reaction volume including: 4.5 μl of sample cDNA, 0.5μl of gene expression assay, and 5μL of Taqman^®^ Gene Expression Master Mix (Applied Biosystems, USA). Each sample was analyzed in triplicate in 96-well plates using the 7500 Real-Time PCR System (Applied Biosystems, USA). The geometric mean of the two closest values for each sample was used for data analysis. On each plate, a standard curve was generated from 1:10 serial dilutions of cDNA transcribed from human universal reference RNA (Stratagene, USA). All samples were also tested using expression assays for two endogenous control HPRT1 (Hs01003267) and 18S (Hs99999901). The relative quantitative expression levels of the tested genes were normalized against the mean value of the endogenous controls [gene expression level = sirtuin mean quantity/ mean (18S and HPRT1 quantity)].

### Immunohistochemistry

SIRT1 and SIRT7 protein expression were studied by IHC. Sections (3μm thick) from the FFPE samples, mounted on glass slides, were deparaffinised in xylene and hydrated through a graded alcohol series. Antigen retrieval was accomplished by microwaving the slides in EDTA buffer (20′ and 40′ for SIRT1 and SIRT7, respectively) and endogenous peroxidase activity was blocked with 0.6% hydrogen peroxide.

Protein detection was performed using the NovolinkTM Max Polymer Detection System (Leica Biosystems, Nussloch, Germany), according to manufacturer instructions. Slides were incubated overnight at 4°C with mouse monoclonal antibodies specific for SIRT1 (#ab32441, 1:750, Abcam, Cambrige, United Kingdom) and SIRT7 (SC-365344, 1:100, Santa Cruz Biotechnology, Dallas, USA). The slides were washed, developed with diaminobenzidine chromogen and counterstained with hematoxylin. Finally, after dehydration and diaphanization, slides were mounted in Entellan^®^ (Merck-Millipore, Germany). Colorectal mucosa and kidney parenchyma sections were used as positive controls for SIRT1 and SIRT7, respectively.

Semi-quantitative assessment of SIRT1 and SIRT7 nuclear protein expression was done using Allred score [32, 33], by estimating the proportion and intensity of positive cells (range 0, 2 to 8). Scores of 3 or greater were defined as positive.

### Western blot analysis

Protein was extracted from fresh-frozen tissue using Kinexus Lysis Buffer (Kinexus Inc., Vancouver, Canada) and subsequently quantified using a Pierce BCA assay (Thermo Scientific Inc., Bremen, Germany), according to the manufacturer's instructions. For Western blot, 30μg of total protein of each sample was loaded in a 10% sodium dodecyl sulfate polyacrylamide gel for electrophoresis (SDS-PAGE). Proteins were blotted onto 0.2μm PVDF membranes (Bio-Rad Laboratories Inc., USA) and incubated overnight at 4°C with primary antibodies for SIRT1 (ab32441, 1:1000, Abcam, Cambridge, UK) and SIRT7 (SC-365344, 1:500, Santa Cruz Biotechnology, Dallas, USA). To ascertain equal loading of protein, the membranes were also probed with a monoclonal mouse antibody against β-Actin (clone AC-15, 1:8000, Sigma-Aldrich, CO., St. Louis, MO). The ClarityTM Western ECL Substrate (Bio-Rad, Hercules, CA, USA) was used to develop the membranes which were then recorded with Amersham Hyperfilm (GE Healthcare Buckinghamshire, UK). Protein band optical densities were determined by scanning and analyzing ECL signals in the linear range using Bio-Rad Image Lab 5.2.1 software. Values were normalized to the level of β-Actin in each sample.

### Statistical analysis

Data was tabulated and analyzed using STATA (STATACorp, Texas, USA). Wilcoxon rank-sum test was used to compare SIRT mRNA expression levels between ECs and NNE. Pairwise comparisons between EC Type I, Type II and NNE were performed and analyzed using Wilcoxon rank-sum test with Bonferroni adjustment. Fisher's exact test was used to compare positive *vs.* negative SIRT IHC expression proportions between ECs and NNE. Kruskal-Wallis test was used to compare differences in Allred score between ECs and NNE, and clinicopathological features. Wilcoxon rank-sum test was used to compare Western-blot protein band densities between ECs and NNE. Spearman's rank correlation coefficient was used to evaluate the correlation between SIRT protein and mRNA expression. A p value equal or inferior to 0.05 was considered significant.
